# Phylogenetic and expression analysis of the angiopoietin-like gene family and their role in lipid metabolism in pigs

**DOI:** 10.5713/ab.23.0057

**Published:** 2023-05-04

**Authors:** Zibin Zheng, Wentao Lyu, Qihua Hong, Hua Yang, Ying Li, Shengjun Zhao, Ying Ren, Yingping Xiao

**Affiliations:** 1 State Key Laboratory for Managing Biotic and Chemical Threats to the Quality and Safety of Agro-products, Institute of Agro-product Safety and Nutrition, Zhejiang Academy of Agricultural Sciences, Hangzhou 310021, China; 2 State Key Laboratory of Animal Nutrition, College of Animal Science and Technology, China Agricultural University, Beijing 100193, China; 3 College of Animal Sciences, Zhejiang University, Hangzhou 310058, China; 4 Guangdong Provincial Key Laboratory of Animal Molecular Design and Precise Breeding, College of Life Science and Engineering, Foshan University, Foshan 528000, China; 5 Hubei Key Laboratory of Animal Nutrition and Feed Science, Wuhan Polytechnic University, Wuhan 430023, China

**Keywords:** Angiopoietin-like Protein, Evolution, Expression, Fat Metabolize, Jinhua Pig

## Abstract

**Objective:**

The objective of this study was to investigate the phylogenetic and expression analysis of the angiopoietin-like (*ANGPTL*) gene family and their role in lipid metabolism in pigs.

**Methods:**

In this study, the amino acid sequence analysis, phylogenetic analysis, and chromosome adjacent gene analysis were performed to identify the *ANGPTL* gene family in pigs. According to the body weight data from 60 Jinhua pigs, different tissues of 6 pigs with average body weight were used to determine the expression profile of *ANGPTL1–8*. The ileum, subcutaneous fat, and liver of 8 pigs with distinct fatness were selected to analyze the gene expression of *ANGPTL3*, *ANGPTL4*, and *ANGPTL8*.

**Results:**

The sequence length of *ANGPTLs* in pigs was between 1,186 and 1,991 bp, and the pig *ANGPTL* family members shared common features with human homologous genes, including the high similarity of the amino acid sequence and chromosome flanking genes. Amino acid sequence analysis showed that *ANGPTL1–7* had a highly conserved domain except for *ANGPTL8*. Phylogenetic analysis showed that each ANGPTL homologous gene shared a common origin. Quantitative reverse-transcription polymerase chain reaction analysis showed that *ANGPTL* family members had different expression patterns in different tissues. *ANGPTL3* and *ANGPTL8* were mainly expressed in the liver, while *ANGPTL4* was expressed in many other tissues, such as the intestine and subcutaneous fat. The expression levels of *ANGPTL3* in the liver and *ANGPTL4* in the liver, intestine and subcutaneous fat of Jinhua pigs with low propensity for adipogenesis were significantly higher than those of high propensity for adipogenesis.

**Conclusion:**

These results increase our knowledge about the biological role of the ANGPTL family in this important economic species, it will also help to better understand the role of ANGPTL3, ANGPTL4, and ANGPTL8 in lipid metabolism of pigs, and provide innovative ideas for developing strategies to improve meat quality of pigs.

## INTRODUCTION

The angiopoietin-like protein (ANGPTL) family is a type of secretory protein that plays a variety of roles in lipid metabolism, glucose metabolism, energy metabolism, angiogenesis, and stem cell biology [1]. Excluding ANGPTL8, ANGPTL families are similar to Angiopoietins (ANGs) and share common characteristics, including the N-terminal signal sequence and coiled-coil domain (CCD), that mediates the formation of homo-oligomers, and the C-terminal fibrinogen domain (FReD) that regulates ligand activity [2]. ANGPTL8, an atypical member of the ANGPTL family, consists of 198 amino acids and lacks FReD in humans [3]. Disordered lipid metabolism will lead to cardiovascular disease, diabetes, and obesity, and it also affects the meat quality of animals [4].

The investigation on improving meat production as well as meat quality has been pursued by many animal husbandry researchers. Extensive studies confirm that ANGPTL3, ANGPTL4 and ANGPTL8 play an important role in lipid metabolism and are all lipoprotein lipase (LPL) inhibitors [5]. LPL was originally identified as a triglyceride (TG) scavenging factor lipase, it is produced by cardiomyocytes and adipocytes and is transported by GPIHBP1 protein to the lumen side of capillary endothelial cells [6], where it hydrolyzes TG into fatty acids for being absorbed into tissues.

ANGPTL3 is a liver-specific secretion factor and is highly expressed in the liver [7]. Its expression is regulated by the liver X receptor (LXR). The main effect of ANGPTL3 is to inhibit the activity of LPL in the capillaries of adipose tissue and muscle [8]. Intriguingly, ANGPTL3 plays a major role in promoting the uptake of very low density lipoprotein (VLDL)-derived TG into white adipose tissue rather than oxidative tissues such as skeletal muscle, heart brown adipose tissue in the feeding state [9].

ANGPTL4 is also known as fasting-induced adipokine (FIAF) or peroxisome proliferator-activated receptor gamma angiopoietin-related protein (PGAR) [10]. ANGPTL4 regulates lipid homeostasis and participates in regulating the intestinal microbiota in fat deposition [11]. Unlike ANGPTL3, ANGPTL4 can be secreted by adipose tissue, intestinal tissues, liver, skeletal muscle, heart, and other tissues [12]. ANGPTL4 is released as a 50 kD hormone precursor, which is then cut into N-terminal and C-terminal fragments, the N-terminus of ANGPTL4 acts as a lipoprotein lipase (LPL) inhibitor [12]. Research shows that ANGPTL4 is negatively correlated with low-density lipoprotein (LDL) cholesterol and high-density lipoprotein (HDL) cholesterol and is positively correlated with various lipid metabolic syndromes [13]. ANGPTL4 is also an endogenous inhibitor to intestinal fatty digestive enzymes. The knockout of ANGPTL4 in mice would increase the body weight, decrease the lipid content in feces, and increase dietary triglyceride accumulation in the small intestine, which was consistent with the increased intestinal lumen lipase activity [14]. ANGPTL3 mainly inhibits LPL activity in the feeding state, whereas ANGPTL4 plays an important role in both feeding and fasting states [15]. Different from ANGPTL3, ANGPTL4 is considered to be an endocrine or autocrine/paracrine inhibitor of LPL due to its different expression sites.

ANGPTL8 is a new protein mainly expressed in the human liver and plays an important role in regulation of plasma TG levels and lipid metabolism [16]. In addition, the N-terminal domain of ANGPTL8 is 20% identical to ANGPTL3 and ANGPTL4 and functionally binds to LPL to regulate triglyceride metabolism, indicating the affinity between them [17]. ANGPTL8 could interact with ANGPTL3, and they could synergistically inhibit LPL activity and regulate the plasma triglyceride levels [18]. Expression of ANGPTL8 in *ANGPTL3* knockout mice failed to increase the levels of triacylglycerol non-esterified fatty acids in plasma [19]. Since ANGPTL3, ANGPTL4, and ANGPTL8 are all LPL inhibitors, the absence of anyone of them would break the balance of triglyceride metabolism in turn would lead to hypotriglyceridemia or hypertriglyceridemia. During fed-fast cycle, feeding could induce the expression of ANGPTL8 and activate the interaction between ANGPTL8 and ANGPTL3 in mice, thereby inhibiting LPL activity in the myocardium and bones. Conversely, fasting could inhibit the expression of ANGPTL8 and improve the expression of ANGPTL4 in mice, resulting the transportation of TG to muscles [20].

So far, studies on the ANGPTLs have focused on mice and humans [21], but reports on the *ANGPTL* gene in domestic animals are limited. The Jinhua pig is an important breed in China with a high percentage of body fat and good meat quality. Pigs are more closely related to humans in carbohydrate and lipid metabolism than mice [22]. Compared to mice, pigs are considered to be more suitable to be developed as animal models for studying human obesity and fat metabolism [23]. In this study, we reported the basic information of the *ANGPTL1–8* gene in Jinhua pigs, the evolutionary relationship between the pig *ANGPTL1–8* gene and other different vertebrates, their expression profile in different tissues, and the differences in the mRNA expression levels of *ANGPTL3*, *ANGPTL4*, and *ANGPTL8* between obese and lean Jinhua pigs. The purpose of this study is to establish a theoretical foundation for the roles of the *ANGPTL* family genes in the fat regulation of pigs.

## MATERIALS AND METHODS

All animals used in this study were reviewed and approved by the Institutional Animal Care and Use Committee of Zhejiang Academy of Agricultural Sciences (2019ZAASLA37). Written informed consent was obtained from the owners for the participation of their animals in this study.

### Data mining and gene identification

The genome portion of pig *ANGPTL* family members were retrieved from the NCBI database to obtain basic information, such as transcript ID and mRNA length (bp) [24]. The relative molecular weights and charge was performed using the programs within the DNASTAR Lasergene [25], the ClustalW was used to generate the amino acid sequences [26]. Exon-intron boundaries and chromosomal locations were identified by the mRNA-genome alignment program Spidey [27].

### Phylogenetic and evolutionary analyses

The MUSCLE program was used to align amino acid sequences (obtained from the NCBI database), and Boxhade software was used for visual display. Phylogenetic analysis of the ANGPTL family members in humans and animals, including mice, cattle, pigs, dogs, and chickens, was carried out using the deduced mature protein sequences. The Mega7.0 software was used to construct phylogenetic trees from P-distances of ANGPTL amino acid sequences from pigs and other representative vertebrates [28]. The Bootstrap method was used to test the reliability of each branch 1,000 times.

### Short-range gene linkage

In order to further confirm the homology of the *ANGPTL* gene and establish the evolutionary model of the *ANGPTL* gene in metazoa, we isolated the genetic environment of ANGPTL3 and ANGPTL4 chromosomes or genomic fragments of vertebrates to determine whether there are homologous genomic regions in vertebrates. Similarly, the genetic environment of mammalian ANGPTL8 is described. The short-range gene linkage comparison was performed among humans, mice, cattle, pigs, dogs, and poultry. The adjacent genetic environment of vertebrates was retrieved from the Genomicus database, and the genetic environment of human ANGPTL3, ANGPTL4, and ANGPTL8 were used as a reference sequence [29]. Homologous genomic regions were characterized by the discovery of specific genomic combinations of conserved flanking genes.

### Animals and sampling

A total of 60 Jinhua Pigs at 90-day old were obtained from the experimental pig farm of Jinhua Academy of Agricultural Sciences (Jinhua, Zhejiang Province, China). All Jinhua pigs were reared in pens (10 pens, 6 pigs per pen) in an environmentally controlled facility and had free access to commercial diets and water under a standard management. At 270 days of age, pigs were weighed individually and slaughtered. Backfat thickness was measured. Based on the body weight data, different tissues were collected from 6 pigs with average body weight, immediately frozen in liquid nitrogen, and stored in a −80°C freezer until RNA isolation. The tissues included tongue, esophagus, stomach, duodenum, jejunum, ileum, cecum, colon, trachea, lung, heart, longissimus muscle, subcutaneous fat, liver, kidney, brain, pancreas, and spleen. Eight of 92.35±10.10 kg pigs with the higher and lower backfat thickness were set as the high group (H group) and low group (L group), respectively [30]. The ileum, subcutaneous fat, and liver segments of the H and L group pigs were collected, immediately frozen in liquid nitrogen, and stored in a −80°C freezer until RNA isolation.

### RNA extraction and real-time quantitative polymerase chain reaction

According to the manufacturer’s instructions, total RNA was extracted from each tissue sample using a TRI-zol Plus RNA Purification Kit (Invitrogen, Carlsbad, CA, USA). RNA integrity and purity were assessed by Nanodrop after being electrophoresed in a formaldehyde gel [31,32]. The genomic DNA contamination was removed from the RNA samples using a gDNA Eraser (Takara Bio Inc., Dalian, China), and the PrimeScript RT reagent kit (Takara Bio Inc., China) was subsequently used according to the manufacturer’s instructions for reverse transcription. The cDNA was then diluted ten times with RNase-free water before quantitative real-time quantitative polymerase chain reaction (RT-qPCR) analysis. RT-qPCR was performed on each sample by using the CFX96 RT-qPCR Detection System (Bio-Rad Laboratories, Richmond, VA, USA), and the amplification was conducted in a total volume of 20 μL, containing 10 μL of SYBR Premix Ex Taq II (Takara Bio Inc., China), 7 μL of RNase-free water, 1 μL of the diluted cDNA, and 0.5 μL of each primer (Table 1). The optimum RT-qPCR program was 95°C for 1 min, followed by 40 cycles of 95°C for 15 s and 60°C for 25 s. Relative quantification of the mRNA transcripts was accomplished using the 2-ΔΔCT method and the housekeeping gene was glyceraldehyde-3-phosphate dehydrogenase (*GAPDH*).

### Statistical analysis of data

Data analysis were performed by SPSS 23.0 (IBM, New York, NY, USA) followed by an unpaired two-tailed Student’s t-test [33]. Relative expression data are presented as mean±standard error of the mean. Statistical significance was considered at p<0.05. The relative expression levels of *ANGPTL3*, *ANGPTL4*, and *ANGPTL8* in different tissues from Jinhua Pigs with different amounts of body fat were performed with the GraphPad Prism version 8.00 (GraphPad Software, LaJolla, CA, USA).

## RESULTS

### Basic information of pig ANGPTL1–8

The nucleotide sequences of pig ANGPTL1–8 were obtained from the NCBI database, and the basic information was generated by DNASTAR Lasergene (Table 1). The mRNA sequences of pig ANGPTL1–8 were between 1,100 and 2,000 bp in length; the numbers of amino acid residues of ANGPTL1–7 were between 344–493 while ANGPTL8 were198. ANGPTL1–8 was composed of 4 to 8 exons determined by Spidy program analysis (Table 2).

### Sequence conservation of pig ANGPTLs

**Figure 1:**
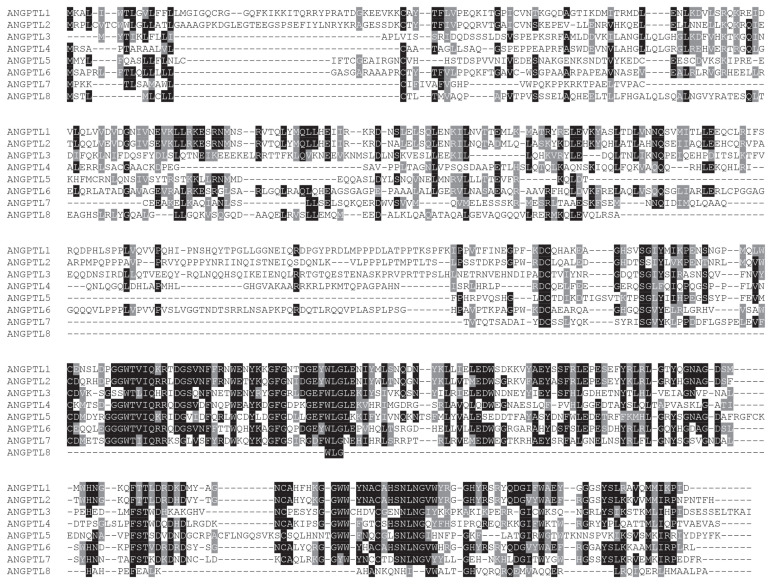
Multiple sequence alignment of representative pig angiopoietin-like (ANGPTL) protein sequences. The conserved residues are shaded. Specifically, the black part is highly similar, the gray part is less similar, and the non-color covered part has no similarity.

Multiple sequence alignment of amino acid sequences of pig ANGPTL1–8 showed that the amino acid sequence of ANGPTL1–7 was highly conserved, and the amino acid sequence of ANGPTL8 was less conserved (Figure 1). Therefore, the structure of ANGPTL8 may be different from other members in the ANGPTL family. According to the Editsequence calculation, amino acid sequences of pig ANGPTL1–8 had 95.93%, 97.57%, 84.13%, 79.37%, 91.75%, 88.35%, 89.28%, and 71.72% homology with the corresponding amino acid sequences of human ANGPTL1–8.

### Phylogeny of the pig ANGPTLs

**Figure 2:**
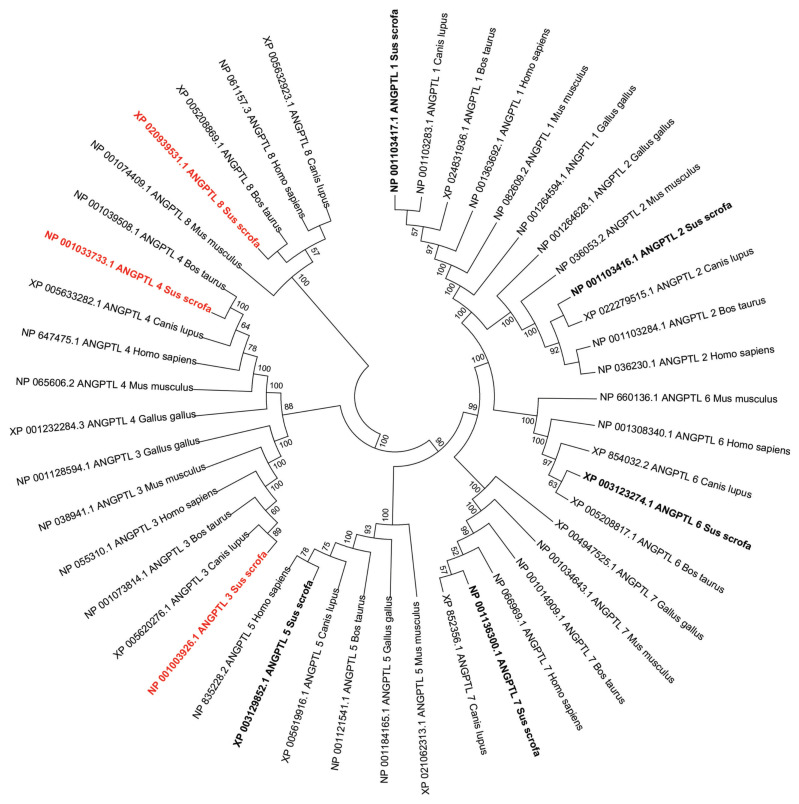
Phylogenetic analysis of pig angiopoietin-like (*ANGPTL*) genes and their representative sequences from human and animals. The topology was constructed using the amino acid sequences by the Neighbor-Joining method with 1,000 bootstrap repeats. Only the branches with a bootstrap value greater than 50 are displayed at branching points.

Phylogenetic analysis of the vertebrate ANGPTL family suggested that these genes shared a common origin (Figure 2). According to the dendritic topology, there are five main protein clusters that contain different members of the ANGPTL family: the ANGPTL1-2-6 cluster (ANGPTL1, ANGPTL2, and ANGPTL6), the ANGPTL3–4 cluster (ANGPTL3, ANGPTL4), the ANGPTL5 cluster, the ANGPTL7 cluster, and the ANGPTL8 cluster, which was isolated from the other four protein clusters (Figure 2). Each ANGPTL orthologous gene was clustered with other mammalian sequences and separately from poultry.

### Neighboring gene analysis

**Figure 3:**
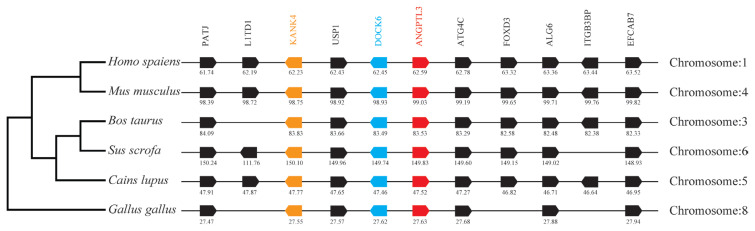
Comparison of homologous genomic regions of pig *ANGPTL3* with humans, mice, and several other animals. The genetic environment of the pig *ANGPTL3* gene was characterized, and the homologous genes of humans, mice, and other animals were identified. The horizontal line represents the chromosome fragments, the arrow box represents the gene, and the arrowhead points in the direction of predicted gene transcription. Only genes conserved across species are displayed. The same color is of the gene homologs, and they are presented according to their order in the chromosome. The gene names and symbols are: *PATJ*, PATJ crumbs cell polarity complex component); *L1TD1*, LINE1 type transposase domain containing 1; *KANK4*, KN motif and ankyrin repeat domains 4; *USP1*, ubiquitin-specific peptidase 1; *DOCK6*, dedicator of cytokinesis 6; *ANGPTL3*, angiopoietin-like 3; *ATG4C*, autophagy related 4C cysteine peptidase; *FOXD3*, forkhead box D3; *ALG6*, ALG6 alpha-1,3-glucosyltransferase; *ITGB3BP*, integrin subunit beta 3 binding protein; *EFCAB7*, EF-hand calcium binding domain 7.

**Figure 4:**
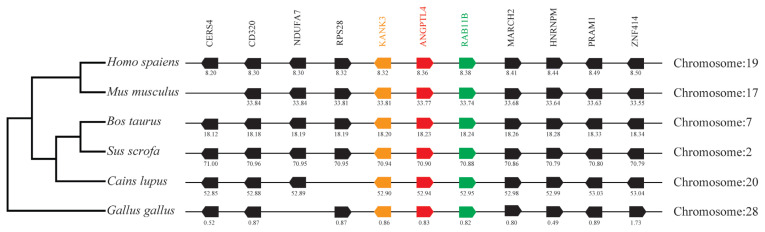
Comparison of homologous genomic regions of pig *ANGPTL4* with humans, mice, and several other animals. The genetic environment of the pig *ANGPTL4* gene was characterized, and the homologous genes of humans, mice, and other animals were identified. The horizontal line represents the chromosome segment, the arrow box represents the gene, and the arrowhead points in the direction of predicted gene transcription. Only genes preserved across species will appear. The same color is of the gene homologs, and they are presented according to their order in the chromosome. The gene names and symbols are: *CERS4*, ceramide synthase 4; *CD320*, CD320 molecule; *DUFA7*, NADH:ubiquinone oxidoreductase subunit A7; *RPS28*, Ribosomal protein S28; *KANK3*, KN motif, and ankyrin repeat domains 3; *ANGPTL4*, angiopoietin-like 4; *Rab11B*, RAB11B, member RAS oncogene family; *MARCH2*, membrane-associated ring finger (C3HC4) 2; *HNRNPM*, heterogeneous nuclear ribonucleoprotein M; *PRAM1*, PML-RARA regulated adaptor molecule 1; *ZNF414*, zinc finger protein 414.

**Figure 5:**
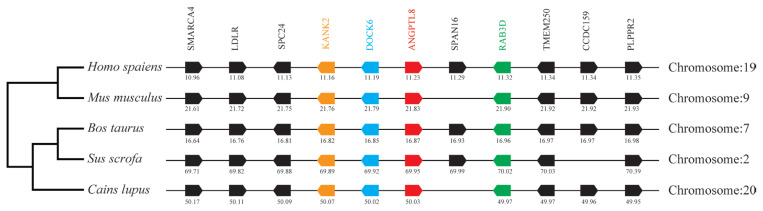
Comparison of homologous genomic regions of pig *ANGPTL8* with humans, mice, and several other animals. The genetic environment of the pig *ANGPTL8* gene was characterized, and the homologous genes of humans, mice, and other animals were identified. The horizontal line represents the chromosome segment, the arrow box represents the gene, and the arrowhead points in the direction of predicted gene transcription. Only genes preserved across species will appear. The same color is of the gene homologs, and they are presented according to their order in the chromosome. The gene names and symbols are: *SMARCA4*, SWI/SNF related, matrix associated, actin-dependent regulator of chromatin, subfamily a, member 4; *LDLR*, low density lipoprotein receptor; *SPC24*, SPC24 component of NDC80 kinetochore complex; *KANK2*, KN motif and ankyrin repeat domains 2; *DOCK6*, dedicator of cytokinesis 6; *ANGPTL8*, angiopoietin-like 8; *SPAN16*, sperm peptide antigen; *RAB3D*, RAB3D, member RAS oncogene family; *TMEM250*, Transmembrane protein 250; *CCDC159*, Coiled-coil domain containing 159; *PLPPR2*, phospholipid phosphatase related 2.

By analyzing and comparing the genetic environment of ANGPTL3 (Figure 3) and ANGPTL4 (Figure 4) in mammals and poultry, we could further understand the evolutionary relationship of ANGPTL family genes in vertebrate radiation. The pig *ANGPTL3* gene was mapped to chromosome 6, and the *ANGPTL4* gene was mapped to chromosome 2 and was also found to be present in other mammals and chickens. Among the ten genes flanking human ANGPTL3, nine were retained on pig chromosome 6, and seven were retained on chicken chromosome 8. Of the ten genes flanking human ANGPTL4, nine were retained on pig chromosome 2, and nine were retained on chicken chromosome 28. This indicated that the genetic environment of several major mammals (humans, mice, cattle, pigs, and dogs) ANGPTL3 was highly similar and was also similar to that of chicken ANGPTL3, indicating that mammals and poultry ANGPTL3 and ANGPTL4 shared a common ancestral origin. Comparing the adjacent genetic environment of mammalian ANGPTL8 (Figure 5) in pigs, ANGPTL8 was located on chromosome 2. Among the ten genes flanking human ANGPTL8, all were retained on cow chromosome 7, and nine genes were retained on pig chromosome 2. This indicated that the genetic environment of ANGPTL8 in several major mammals (humans, mice, cattle, pigs, dogs) were highly similar, which means that the mammalian ANGPTL8 had a common ancestral origin.

### Gene expression pattern of pig ANGPTL family

**Figure 6:**
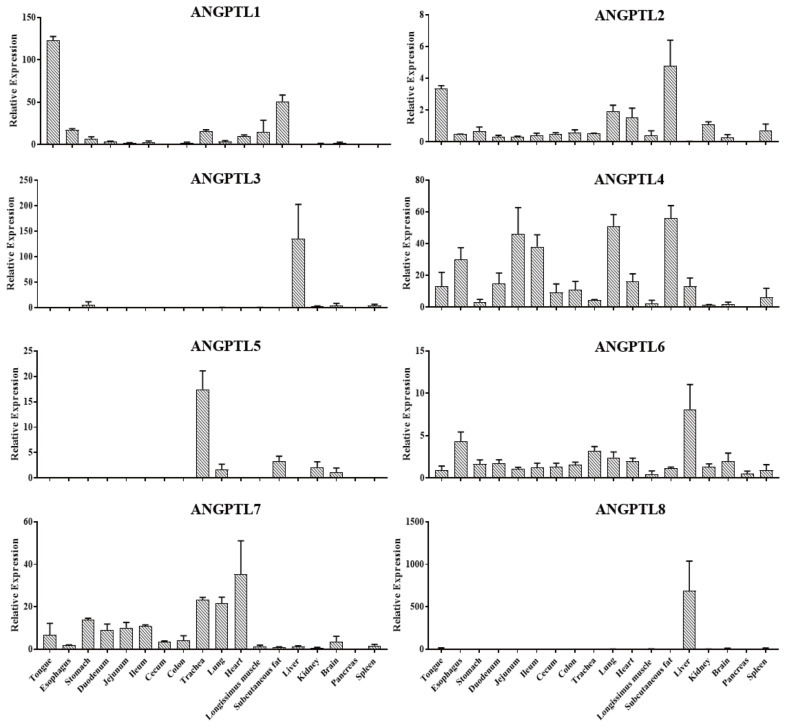
Relative expression of *ANGPTL1–8* in different tissues, including tongue, esophagus, stomach, duodenum, jejunum, ileum, cecum, colon, trachea, lung, heart, longissimus muscle, subcutaneous fat, liver, kidney, brain, pancreas, and spleen of Jinhua pigs. The indicated tissue segments were collected from 6 Jinhua pigs at 270 days of age following by RNA isolation, reverse transcription and RT-qPCR. *ANGPTL8*, angiopoietin-like 8. Data was expressed as mean±standard error of the mean (n = 6).

In order to study the expression patterns of the *ANGPTL* gene family in pigs, the mRNA abundance of ANGPTL1–8 in 18 tissues of 6 Jinhua pigs was determined by RT-qPCR. As shown in Figure 6, ANGPTL4 was widely expressed in various tissues, while ANGPTL3 and ANGPTL8 were predominantly expressed in the liver, and ANGPTL8 was only expressed in the liver. Meanwhile, it was observed that ANGPTL4 was highly expressed in the intestinal tissues with relative low expression in subcutaneous fat, lung, heart, and longissimus muscle. ANGPTL4 relative expression level was the highest in subcutaneous fat.

### Expression levels of *ANGPTL3*, *ANGPTL4*, and *ANGPTL8* in Jinhua pigs with distinct fatness

**Figure 7:**
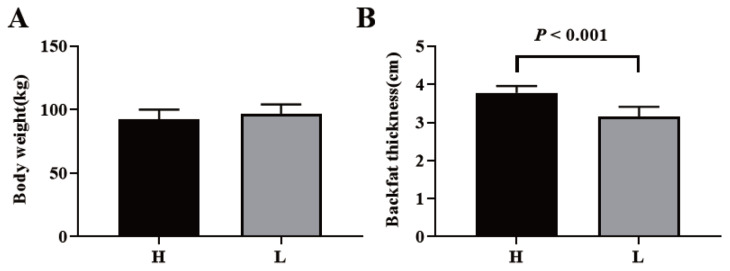
Body weight (A) and backfat thickness (B) of Jinhua pigs in the H and L groups. Jinhua pigs with similar body weight and significantly different backfat thickness were weighed individually and the backfat thickness of each pig was measured at 270 days of age. H, the H group consisted of pigs with relatively high backfat thickness; L, the L group was consisted of pigs with relatively low backfat thickness. *ANGPTL*, angiopoietin-like. Data was expressed as mean±standard error of the mean (n = 8) and analyzed by one-way analysis of variance analysis followed by an unpaired two-tailed student’s t-test.

**Figure 8:**
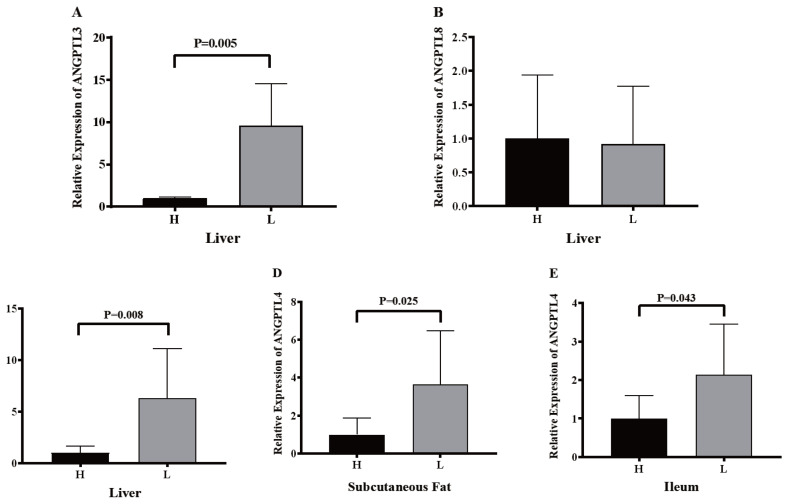
The expression of *ANGPTL3*, *ANGPTL4*, and *ANGPTL8* in the liver (A, B, and C), subcutaneous fat (D), and ileum (E) of Jinhua pigs in the H and L groups. The liver, subcutaneous fat, and ileum segments were collected from 16 Jinhua pigs at 270 days of age following by RNA isolation, reverse transcription and RT-qPCR. H, the H group consisted of pigs with relatively high backfat thickness; L, the L group was consisted of pigs with relatively low backfat thickness. Data was expressed as mean±standard error of the mean (n = 8) and analyzed by one-way analysis of variance analysis followed by an unpaired two-tailed student’s t-test.

To confirm whether lipid metabolism was associated with the expression of *ANGPTL3*, *ANGPTL4*, and *ANGPTL8*, eight of 92.35±10.10 kg pigs with the higher and lower backfat thickness were set as the high group (H group) and low group (L group). Pig *ANGPTL3*, *ANGPTL4*, and *ANGPTL8* were analyzed by RT-qPCR following RNA isolation and reverse transcription. The backfat thickness of Jinhua pigs in the H group was significantly higher than that of Jinhua pigs in the L group (Figure 7). However, there were no significant differences in the body weight of Jinhua pigs between the H and L groups (Figure 7). The relative expression of *ANGPTL3* in the liver of the H group pigs was significantly lower than that of the L group pigs (p = 0.005) while the relative expression levels of *ANGPTL4* in the ileum (p = 0.043), subcutaneous fat (p = 0.025), and liver (p = 0.008) of the H group pigs were lower than those of the L group pigs (Figure 8). However, the relative expression of *ANGPTL8* in the liver of the H group pigs was not significantly different from that of the L group pigs (p>0.05).

## DISCUSSION

The Jinhua pig has become one of the most important local breeds in China due to its excellent meat quality and it exhibits the characteristics of early sexual maturity, low prolificacy, and a high body fat content [34]. The purpose of this study was to identify the phylogenetic and expression analysis of ANGPTL family genes in Jinhua Pig, a model animal of lipid metabolism, and the important roles of ANGPTL3, ANGPTL4, and ANGPTL8 in lipid metabolism. In the present study, amino acid sequence analysis, phylogenetic analysis, chromosome adjacent gene analysis, and RT-qPCR analysis showed that Jinhua pig ANGPTL family members had common characteristics with homologous human genes, including the high similarity of amino acid sequence and chromosome flanking genes. Consistent with earlier studies, pig ANGPTL3–4 had typical angiopoietin family structures. The structures of pig ANGPTL3–4 is similar to those of humans and mice, and both had FReD, which affects angiogenic activity, and CCD, which regulated fat metabolism by inhibiting LPL [35,36]. The eight ANGPTL family member genes in Jinhua pigs were 1,100 to 2,000 bp in length and consisted of 4 to 8 exons. The functional helical domain and fibrinogen-like domain in the ANGPTL1–8 amino acid sequence were strongly conserved. Through the identification and expression analysis of the bovine *ANGPTL1–7* genes, all of the deduced amino acid sequences had an N-terminal crimped domain and a C-terminal fibrinogen-like domain, and both had the characteristics of angiogenin. The bovine ANGPTL1–7 amino acid sequences are similar to those of humans, and the corresponding members had 95%, 97%, 82%, 85%, 87%, 85%, and 87% homology [27]. Related studies reported the cloning, chromosomal location, and expression analysis of pig ANGPTL3 and ANGPTL4. The deduced amino acid sequence of pig ANGPTL3 had 83% and 73.7% homology to humans and mice, respectively. While pig ANGPTL4 had 79.4% and 79.1% homology with humans and mice [36]. Homologs of the mammalian *ANGPTL8* gene are not present in sequenced genomes of fish and other non-mammalian vertebrates, ANGPTL8 is an atypical family member that lacks the FReD structural domain, glycosylation sites and amino acids that form intramolecular disulfide bonds, leading to a lower homology of ANGPTL8 with humans in other species [37]. This research showed that pig ANGPTL1–8 amino acid sequence had 95.93%, 97.57%, 84.13%, 79.37%, 91.75%, 88.35%, 89.28%, and 71.72% homology with corresponding human members, respectively.

In vertebrates, the ANGPTL family of genes evolved by gene duplication, gene deletion, and gene mutation [37]. They share the same evolutionary origin with angiogenin and share similarities in sequence and structure [37]. ANGPTL family members expanded in early vertebrate genome doubling and genome segment replication before vertebrate radiation. Five major ANGPTL vertebrate protein clusters, ANGPTL1-2-6, ANGPTL3–4, ANGPTL5, ANGPTL7, ANGPTL8, derived from the replication of ancestor ANGPTL [38]. The ANGPTL8 cluster was isolated from the other four protein clusters since ANGPTL8 has a different structure from other ANGPTL family members [19]. Clustering in phylogenetic trees indicates that pig sequences are not significantly different from those of other vertebrates.

Recent studies have shown that ANGPTL3, ANGPTL4, and ANGPTL8 are directly related to lipid metabolism and fat deposition in human and mice [39]. In this study, tissue expression profiles showed that ANGPTL3 and ANGPTL8 were mainly expressed in the liver of pigs, with similar results in humans and mice [5,40]. However, ANGPTL4 in pigs is widely expressed in many tissues, including the gastrointestinal tract, subcutaneous fat, liver, muscles, heart, and lungs. In humans and mice, ANGPTL4 is also expressed mainly in white adipose tissue and the liver as well as in the intestine and heart tissue [10,41]. By analyzing the expression levels of ANGPTL3 and ANGPTL8 in the liver and the relative expression levels of ANGPTL4 in the liver, subcutaneous fat, and ileum of Jinhua pigs with distinct fatness, we found that the expression of ANGPTL3 in the liver of Jinhua pigs in the H group was significantly lower than that of Jinhua pigs in the L group (p = 0.005). Moreover, the expression level of ANGPTL4 in the liver (p = 0.008), subcutaneous fat (p = 0.025), and ileum (p = 0.043) of the H group pigs was significantly lower than that of the L group pigs. This might be due to the inhibitory effect of ANGPTL3 and ANGPTL4 on LPL. With the decrease in the expressions of ANGPTL3 and ANGPTL4, the activity of LPL would increase, which in turn would decrease the serum TG level and increase the lipid absorption. Early research showed that in sterile mice with routine feeding, the microbiota promoted the storage of triglycerides in fat cells by inhibiting the intestinal expression of ANGPTL4, illustrating the decrease of ANGPTL4 expression is beneficial to fat storage [11]. Recent studies have indicated that the level of circulating ANGPTL4 in obese children was lower than that of normal weight children. Waist circumference, body weight, and other major obesity indicators were negatively correlated with the level of circulating ANGPTL4 [42]. Meanwhile, mice with ANGPTL3 and ANGPTL4 deficiency had severe hypertriglyceridemia while mice with ANGPTL3 overexpression had hyperlipidemia [43]. Furthermore, compared with other breeds of pigs, the fat percentage of Large White pigs was lower with a higher expression of ANGPTL4 mRNA [30]. These studies are consistent with the results of the present study.

Although there was no significant difference in ANGPTL8 expression between the liver of Jinhua pigs in the H and L groups, it might result from the function of ANGPTL8, which promotes the cleavage of ANGPTL3 and binds to the N-terminal of ANGPTL3 [20]. Related studies have confirmed that expression of ANGPTL8 in ANGPTL3 knockout mice failed to promote hypertriglyceridemia [19]. Additionally, the overexpression of ANGPTL8 in the liver of *ANPTL3* gene knockout mice had no effect on the triglyceride metabolism [19]. The interaction of ANGPTL8 with ANGPTL3 would form a complex with the N-terminal of ANGPTL3, synergistically inhibiting the LPL activity and modulating the plasma triglyceride levels [44].

Fat metabolism and fat storage are closely related to the meat quality and carcass quality. Relevant research showed that by comparing the carcass composition and development patterns of Jinhua and Landrace pigs at 35 to 125 days of age, the carcass fat content of Jinhua pigs was significantly higher than that of Landrace pigs (p<0.05), and the lean meat percentage of the p carcass was significantly lower than that of Landrace pigs (p<0.01) [45]. Similar studies showed that Jinhua pigs had a higher tendency to deposit fat compared with Landrace pigs [23]. These are consistent with the results of this experiment. Therefore, Jinhua Pigs with high body fat percentage and good meat quality play an important role in the study of lipid metabolism and its relationship with meat quality.

## CONCLUSION

Overall, the present study identified the basic information and phylogenetic patterns of porcine ANGPTL family gene members, and the expression of ANGPTL1–8 in Jinhua pigs was tissue-specific. This study enriches our understanding of the porcine ANGPTL family genes and confirm the important relevance of ANGPTL3 and ANGPTL4 in regulating lipid metabolism and improving pork quality, heralding a new research direction and molecular theoretical basis for improving pork quality.

## Figures and Tables

**Table 1:** Primers sequences used in the RT-qPCR analysis

Gene	Genebank accession	Primer sequences (5′to3′)	Size (bp)
Pig *ANGPTL1*	NM_001109947.1	GGCAACTCCTCCCACCAAA	106
		GACTGAATGTCCAGCTTCTTTTGCA	
Pig *ANGPTL2*	NM_001109946.1	GGCTCCGTCAACTTCTTTAGG	123
		CAGTTTGTAGTTGCCTTGGTTCGT	
Pig *ANGPTL3*	NM_001003926.2	GCACTCCCAGAACACGAAGA	96
		CCACCAGCCTCCTGAATAACT	
Pig *ANGPTL4*	NM_001038644.1	GACTGCCAAGAGCTGTTTGAAGA	126
		CTGAATTACAGTCCAGCCTCCAT	
Pig *ANGPTL5*	XM_003129804.4	CCAAAGGCTGTGGTGTGATTATC	140
		CATCTTCAGATTCCAGGGCTACA	
Pig *ANGPTL6*	XM_003123226.5	GTATCAGCATGGTGCGAGCAA	99
		GTGCTGCCAAGTAGTGAAGAAGT	
Pig *ANGPTL7*	NM_001142828.2	CTGGAGGTTTTCTGCGACATG	116
		CCGAAGCCTTGCTTGTACTGT	
Pig *ANGPTL8*	XM_021083871.1	GGACCTGTCACGCACCAA	78
		CCGATCCCAGCCAACAG	
Pig *GAPDH*	NM_001206359.1	CCAGGGCTGCTTTTAACTCTG	104
		GTGGGTGGAATCATACTGGAACAT	

*ANGPTL*, angiopoietin-like; *GAPDH*, glyceraldehyde-3-phosphate dehydrogenase.

**Table 2:** Basic information of pig ANGPTL1–8

Name	Transcript ID	mRNA length (bp)	Protein (aa)	Relative molecular mass	Exon no.	Chromosome	Negatively charged residues (Asp + Glu)	Positively charged residues (Arg + Lys)
ANGPTL1	NM_001109947.1	1,856	491	56,582.68	4	9	57	61
ANGPTL2	NM_001109946.1	1,991	493	56,952.39	4	1	55	56
ANGPTL3	NM_001003926.2	1,453	462	53,658.25	7	6,	67	54
ANGPTL4	NM_001038644.1	1,833	412	45,545.53	7	2	39	47
ANGPTL5	XM_003129804.4	1,747	388	44,536.36	8	9	48	42
ANGPTL6	XM_003123226.5	1,849	463	50,726.18	5	2	43	53
ANGPTL7	NM_001142828.2	1,186	344	39,394.58	6	6	41	44
ANGPTL8	XM_021083872.1	1,884	198	21,979.25	4	2	19	16

ANGPTL, angiopoietin-like.

## Data Availability

The datasets reported in this manuscript are available from the corresponding author on reasonable request. The genome portion of pig ANGPTL family members were retrieved from the NCBI database to obtain basic information. The MUSCLE program was used to align amino acid sequences (obtained from the NCBI database), and Boxhade software was used for visual display. The ANGPTL1–8 gene sequence was obtained in NCBI, and their genebank accession numbers are NM_001109947.1, NM_001109946.1, NM_00100 3926.2, NM_001038644.1, XM_003129804.4, XM_00312 3226.5, NM_001142828.2, and XM_021083871.1 respectively.
